# ChromInst: a multicentre evaluation of robustness in aneuploidy and structural rearrangement testing

**DOI:** 10.1186/s12967-025-06242-7

**Published:** 2025-02-26

**Authors:** Wenbin Niu, Shanjun Dai, Linli Hu, Yao He, Xiqian Zhang, Xia Xue, Li Wu, Haixia Jin, Dun Liu, Keya Tong, Senlin Shi, Li Tian, Yifan Zhou, Guidong Yao, Sijia Lu, Yaxin Yao, Dunmei Zhao, Lei Jin, Haiyan Bai, Fenghua Liu, Dongyun Liu, Yingpu Sun

**Affiliations:** 1 Center for Reproductive Medicine, Henan Key Laboratory of Reproduction and Genetics, The First Affliated Hospital of Zhengzhou University, Zhengzhou, China; 2 Chongqing Key Laboratory of Human Embryo Engineering, Center for Reproductive Medicine, Women and Children’s Hospital of Chongqing Medical University, Chongqing, China; 3 Reproductive Medical Center, Guangdong Women and Children Hospital, Guangzhou, China; 4 The Assisted Reproduction Center, Northwest Women’s and Children’s Hospital, Xian, China; 5 Center for Reproductive Medicine, Tongji Hospital, Tongji Medicine College, Huazhong University of Science and Technology, Wuhan, China; 6 Yikon Genomics Co., Ltd., Shanghai, China

**Keywords:** Preimplantation genetic testing for aneuploidy (PGT-A), Preimplantation genetic testing for chromosomal structural rearrangement (PGT-SR), ChromInst, Robust, Accuracy

## Abstract

**Background:**

Preimplantation genetic testing for aneuploidy and for chromosomal structural rearrangement (PGT-A/-SR) can improve clinical pregnancy rates and live birth rates, and shorten the time to pregnancy. The large-scale statistics on their efficacy and accuracy across different centres, as well as the frequency of abnormalities for each chromosome, will provide a valuable supplement to previous research.

**Methods:**

Patients who had PGT-A or -SR procedures at five reproductive centres from 2018 to 2022 were recruited based on PGT-A/-SR indications. ChromInst and next-generation sequencing (NGS)-based PGT technology were utilised to detect copy number variations in embryos. Sequencing data metrics such as median absolute pairwise difference (MAPD) and detection success rate were analysed to evaluate the robustness of ChromInst. To assess ChromInst’s accuracy, the chromosomal results from amniocentesis, abortions, and neonatal blood was as the gold standard for negative PGT results; the fluorescence in situ hybridisation (FISH), which was performed on embryos that identified as aneuploid through PGT was as the gold standard for positive PGT results. The frequency of abnormalities in each chromosome was also explored in aneuploid embryos.

**Results:**

A total of 5,730 embryos were tested from 1,015 patients in the study, 391 of whom had PGT-A and 624 of whom had PGT-SR. 99.5% (5,699/5,730) of the embryos had an NGS sequencing MAPD value < 0.25, and 99.3% (5,689/5,730) of the embryos achieved successful PGT-A/-SR detection. Compared with the gold standard, the concordance of negative PGT-A/-SR results was 99.8% (506/507), and that of positive results was 99.8% (1,123/1,125). The euploidy rate in the PGT-A population was 45.9% (981/2,135). The proportion of euploid + balanced embryos was highest among couples with non-polymorphic inversions (44.6%, 152/341), followed by those with Robertsonian translocations (39.0%, 293/752), and lowest among those with reciprocal translocations (22.5%, 483/2,143). Chromosomes 16, 22, and 15 had the highest frequency of autosomal trisomies among the embryos from PGT-A patients, while chromosomes 16, 22, and 21 had the highest frequency of monosomies. High-frequency chromosomes with de novo chromosomal abnormalities for trisomies and monosomies were similar in the PGT-SR patients to those in the PGT-A patients.

**Conclusions:**

ChromInst-based PGT-A/-SR could accommodate operational variations among different clinical centres, ensuring accurate results through robust and stable detection performance. Prior to PGT-A/-SR, more trustworthy data could be provided to support the genetic counselling.

**Supplementary Information:**

The online version contains supplementary material available at 10.1186/s12967-025-06242-7.

## Background

In vitro fertilisation (IVF) implantation failure and miscarriages are frequently caused by embryonic aneuploidy [1, 2]. Aneuploidy results from errors in gamete meiosis or mitotic errors at different stages of embryonic development [3–5]. The probability of gametogenesis abnormalities increases in populations with chromosomal abnormalities, advanced maternal age, recurrent miscarriage/implantation failure, and sperm abnormalities, leading to a higher rate of embryonic aneuploidy [6–8]. Morphological grading is a method for selecting embryos, but it does not reflect the embryos’ genetic characteristics [9, 10]. Consequently, chromosomal ploidy in embryos for advanced maternal age, recurrent implantation failure, recurrent miscarriage, and severe teratozoospermia is frequently detected clinically using preimplantation genetic testing for aneuploidies (PGT-A) [11, 12]. Preimplantation genetic testing for structural rearrangements (PGT-SR) is used for couples where one or both partners have chromosomal structural abnormalities. These couples frequently have a high rate of natural miscarriages and are prone to an uneven distribution of genetic material during meiosis, which can result in chromosomal abnormalities in embryos [13].

Next-generation sequencing (NGS)-based PGT-A/-SR is a complex technical process that involves multiple steps, including embryo biopsy, sample lysis, whole-genome amplification, library preparation, sequencing, and data analysis, to obtain the final results. The embryo biopsy is performed in the embryology laboratory by embryologists under a microscope. All subsequent testing steps are carried out in the molecular testing laboratory by technicians using specific test reagents and following specific operational requirements. Variances in each of these steps can cause fluctuations in the test results. In the real world, there are variations among embryology labs in terms of the embryo culture medium used, the manufacturers and characteristics of embryo biopsy equipment, the individual habits and proficiency of embryologists, and the liquid carryover volumes during biopsy sample tubing. All of these variances pose challenges to achieving high-quality and stable results in PGT-A [14]. Due to the critical and delicate impact of embryo culture techniques and biopsy procedures on the subsequent viability of embryos, an embryology lab, once it has established its own standard operating procedures (SOPs), is usually unwilling to make any alterations for giving in subsequent assays. This requires the PGT-A/-SR protocol to have high robustness to adapt to variances between laboratories and achieve stable and accurate results. A PGT-A protocol called ChromInst was created in the study [15]. This methodology greatly streamlines the operational processes by integrating NGS library preparation and single-cell whole genome amplification (WGA) into the experimental procedure. In addition to lowering the likelihood of mistakes and omissions, it also improves performance while cutting down on operating time. However, comprehensive evidence confirming its robustness across many centres is still lacking.

Moreover, one of the main issues with the clinical use of PGT in assisted reproduction is false-positives and false-negatives [16, 17]. Viable embryos may be wasted and patients may lose out on implantation opportunities as a result of a false positive PGT result. On the other hand, transferring an embryo with a false negative result may cause unsuccessful implantation, miscarriage, or fetal developmental abnormalities. Zhai et al. conducted NGS-based PGT-SR on embryos from patients with balanced translocations. Amniocentesis was performed on 15 patients who achieved ongoing pregnancies after transferring euploid embryos, and karyotyping results were 100% consistent with PGT-SR results [18]. Nevertheless, the study had a small sample size and did not compare the aneuploid results to the gold standard. A larger sample size and extensive validation of PGT-A/-SR results across several centres are therefore required, particularly for the validation of positive PGT-A/-SR results.

For patients undergoing PGT-A/-SR, the euploidy rate of embryos and the frequency of aneuploidy for each chromosome are critical metrics for assessing embryo development and guiding informed decisions on embryo transfer. Some studies have analysed chromosomal abnormalities in chorionic villus sampling and spontaneous abortions [19, 20]. Ogur et al. analysed 300 couples with structural rearrangements and over 100,000 chromosomal pairs, highlighting the significant impact of rearrangement type, female age (≥ 35 years), and carrier sex on transferable embryo rates. The study found negligible evidence of inter chromosomal effect (ICE) and minor differences in aneuploidy rates between carriers and controls [21]. These findings emphasize the need for personalized genetic assessments for structural rearrangement carriers and further large-scale research to refine clinical applications. To evaluate both the value of the genetic and clinical data and the validation of the technique, this study was conducted. A total of 1,150 infertile couples who underwent PGT-A/-SR-assisted reproduction at five reproductive centres between 2018 and 2022 were recruited. Patients who underwent single euploid blastocyst transfers had their clinical results monitored. The robustness of the ChromInst method was evaluated using sequencing metrics, including median absolute pairwise difference (MAPD) values were used to evaluate the robustness of the ChromInst technique. Using the chromosomal findings of amniocentesis, abortions, and neonatal blood as the negative gold standard and the fluorescence in situ hybridisation (FISH) results of embryo biopsy cells as the positive gold standard, the accuracy of PGT-A/-SR results was confirmed. In order to provide more precise data support for genetic counselling, we additionally examined the euploidy rate and the prevalence of specific chromosomal abnormalities in the PGT-A/-SR population.

## Methods

### Study design and patients

This was a large-scale, multicentre, real-world clinical study. The patients were those who underwent PGT-A/SR from 2018 to 2022 at five reproductive centres: the First Affiliated Hospital of Zhengzhou University, Women and Children’s Hospital of Chongqing Medical University, Guangdong Women and Children Hospital, Northwest Women’s and Children’s Hospital, and Tongji Hospital affiliated with Tongji Medical College of Huazhong University of Science and Technology. Inclusion criteria were female patients undergoing intracytoplasmic sperm injection (ICSI) with at least two usable blastocysts in the first-time oocytes retrieval, and meeting any of the following conditions:

1. Chromosomal structural rearrangement in either or both partners;
2. Having a child with chromosomal abnormalities or chromosomal abnormalities detected in chorionic villus tissue following a miscarriage;
3. ≥3 failed in vitro fertilization-embryo transfer (IVF-ET);
4. ≥2 spontaneous miscarriages;
5. Female aged ≥ 38 years;
6. Severe teratozoospermia.

Exclusion criteria included poor ovarian response; systemic diseases that are clinically significant and unsuitable for pregnancy; simultaneous participation in other clinical studies; and other conditions deemed unsuitable for inclusion by the researchers. The study was approved by the relevant committees at each participating centre (No. Device-2018-05), and all patients signed informed consent forms agreeing to participate in the study.

Depending on their indications, patients received either PGT-A or PGT-SR. The sequencing data was used to analyse the ChromInst’s robustness. The results from amniocentesis, neonatal blood samples, or abortions, using these as the gold standard for negative PGT results, were gathered. For aneuploidy embryos with PGT results, FISH testing was used as the gold standard. Furthermore, the euploidy rate of embryos was calculated, and statistical analysis was performed on the high-frequency abnormal chromosomes in aneuploid embryos.

### ART treatments

Oocyte retrieval must be performed 36 h (± 2) post-hCG administration. Under intravenous anesthesia, oocytes were collected using a 17G aspiration needle guided by transvaginal ultrasound. Post-retrieval, cumulus-oocyte complexes were meticulously identified under a stereomicroscope. The cumulus-oocyte complexes were then collected and washed using a Pasteur pipette and transferred into culture medium. They were incubated at 37 °C in a 5% or 6% CO_2_ incubator until fertilization.

ICSI would be performed as previously described. Briefly, after the cumulus-oocyte complexes were digested with hyaluronidase, the denuded oocytes were assessed for integrity and maturity. Only oocytes that were at the metaphase II stage and had extruded the first polar body were selected for ICSI.

Using the Gardner grading system, embryos were morphologically evaluated on days 5–6 of in vitro culture [22]. A usable blastocyst from approximately 3–5 TE cells spaced apart from the inner cell mass was used for the trophectoderm (TE) biopsy. The biopsy sample was subsequently subjected to genetic testing. After a TE biopsy, the blastocysts underwent vitrification.

### Whole genome amplification, library preparation, and sequencing

For the collected cells, WGA and library preparation were performed using the ChromInst™ (Xukang Medical Technology (Suzhou) Co., Ltd) library kit according to the manufacturer’s instructions [23]. The principle of whole genome amplification is based on the multiple annealing and looping-based amplification cycles (MALBAC) method. Briefly, biopsy cells were lysed, and DNA was annealed with a library of random primers. After pre-amplification, exponential amplification was carried out until 2 µg of DNA was obtained. The amplified product was then used for library preparation with a library construction kit. Quality control of the NGS library was performed using Qubit 3.0 and 1.5% agarose gel electrophoresis. Sequencing was conducted on the Ion Torrent platform (Thermo Fisher Scientific, Waltham, MA, USA), yielding approximately 2 million reads per library.

### Copy number variation (CNV) analysis and MAPD value calculation

CNV analysis and visualization were performed using R packages ‘DNACopy’. High-quality reads were counted in bins of 400 K across the entire genome. These reads were normalized using GC content and a reference dataset. The circular binary segmentation (CBS) algorithm was used to detect CNV segments. Sequencing data that met quality control QC standards and output CNV results were considered successful for PGT-A/-SR detection. The size of segmental aneuploidies was > 10 Mb.

MAPD = median (| X_i+1_ − X_i_|, i ordered by genomic position) was used to calculate the MAPD value of each sample in this study. The MAPD value represented the height difference between two adjacent bins. The smaller the value, the better the uniformity of the scatter plot, and vice versa.

### FISH validation

TE cells from each blastocyst underwent fixation using hypotonic medium (1% sodium citrate in 6 mg/ml bovine serum albumin) [24]. Briefly, the isolated TE cells were placed into droplets of pre-warmed hypotonic medium with incubation time adjusted based on the degree of cell swelling. The swollen cells were then transferred onto a marked area of a coated slide and fixed with a methanol/acetic acid (3:1) solution. Finally, the slides were air-dried at room temperature [24, 25].

Following denaturation at 75 °C, the slides were incubated in a humidified chamber at 37 °C for 16–18 h. After hybridisation, they were carefully washed, counterstained, and finally observed under a fluorescence microscope. For each embryo, FISH analysis targeted the abnormal chromosomes identified by PGT-A/-SR, along with chromosomes 13, 16, 18, 21, X, and Y (Vysis, Abbott Molecular). Additional details of the FISH procedure can be found elsewhere [26].

For each embryo, the target chromosomes for detection are the abnormal chromosomes identified by PGT-A, as well as chromosomes 13, 16, 18, 21, X, and Y.

### Clinical outcomes

The primary clinical outcome was the cumulative live birth rate. A live birth was defined as the delivery of a live infant at a gestational age of more than 28 weeks. The cumulative live birth rate was calculated as the number of cycles resulting in live births divided by the number of oocyte retrieval cycles, including all transfer results within one year post-oocyte retrieval [27].

### Statistical analyses

Statistical analyses were performed using SPSS, version 25.0 (SPSS, Chicago, Illinois, USA). Initially, the normality of the data distribution was assessed. Normally distributed data were presented as mean and standard deviation, while non-normally distributed data were presented as median (Q1-Q3). Categorical variables were expressed as frequencies or percentages. The results of PGT-A/-SR were compared with the results of amniocentesis, newborn peripheral blood, or the FISH, which served as the gold standard. Sensitivity, specificity, positive predictive value (PPV), negative predictive value (NPV), and concordance rate were calculated, with 95% confidence intervals estimated using the Wilson score method.

## Results

**Fig. 1:**
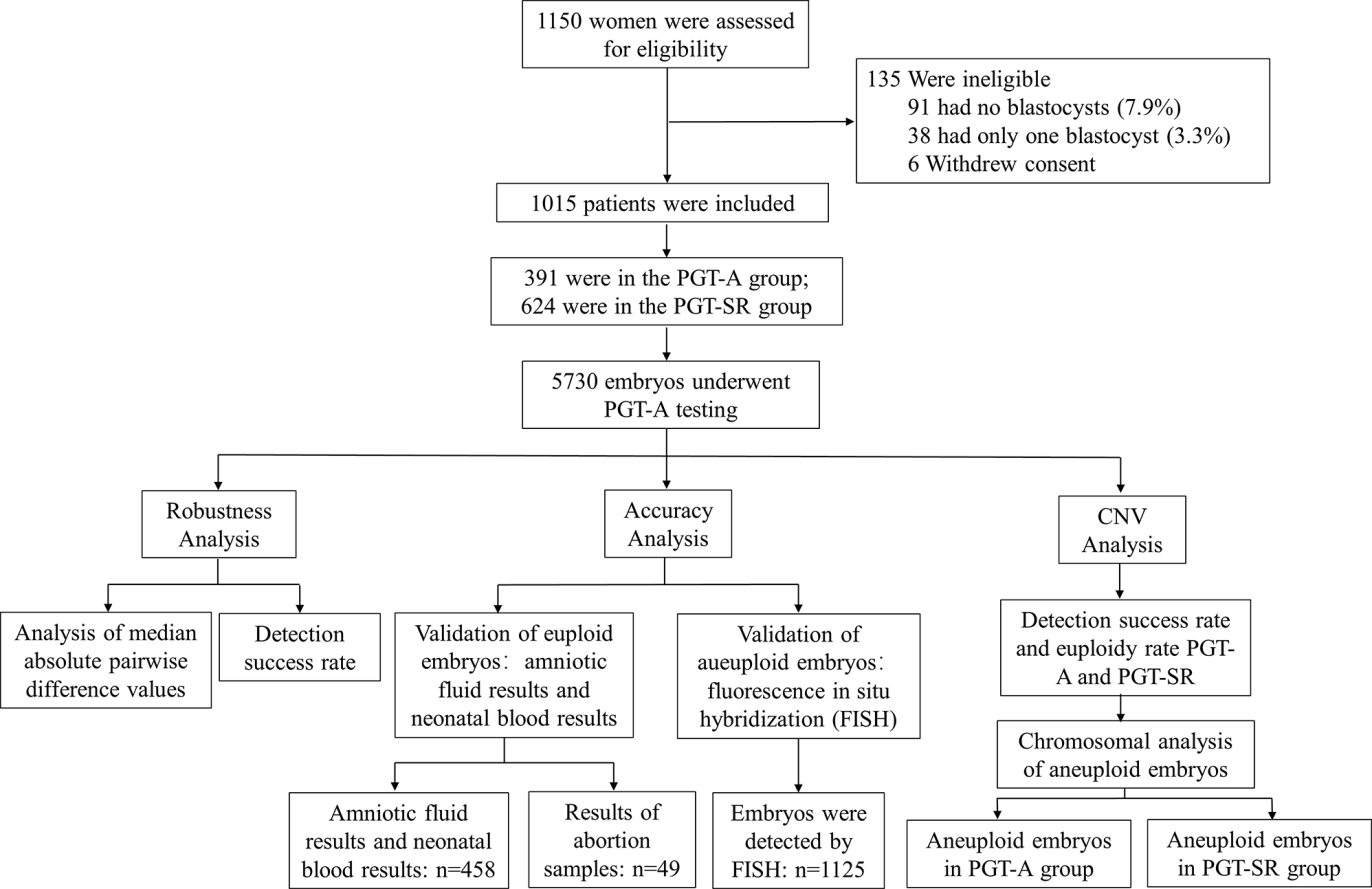
Study flowchart. A total of 1,150 patients were assessed, of whom 1,015 met the inclusion criteria. Among these, 391 patients underwent PGT-A and 624 patients underwent PGT-SR. PGT-A: Preimplantation genetic testing for aneuploidy; PGT-SR: preimplantation genetic testing for structural rearrangements; CNV: copy number variation; FISH: fluorescence in situ hybridisation

A total of 1,150 patients were enrolled in compliance with the PGT indications. A final cohort of 1,015 patients was obtained after 135 patients were excluded for not meeting the inclusion criteria. 5,730 blastocysts in all underwent PGT-A or PGT-SR (Fig. 1).

### Robustness validation of Chrominst

There were differences in embryo culture protocols and biopsy methods across different centres (Supplementary Table 1), and these differences may affect the efficiency of whole-genome amplification. The MAPD is a key indicator for assessing the quality of single-cell CNV and PGT-A/-SR. Osman and Scott et al. recommended an MAPD value of < 0.25 as an acceptable standard for detection quality [28].

To ensure quality control of the test results, each centre performed parallel operations using negative (euploid cells) and positive (trisomy 21 cells) control samples alongside each batch of clinical samples. A total of 534 reference samples were tested across five centres, with each centre testing 178, 126, 98, 52, and 80 reference samples, respectively (Table 1). The negative and positive reference samples were uniformly prepared in vitro cultured cell line samples, each containing three cells.

**Table 1:** MAPD value and detection success rate from each centre

Centre	Samples	Number	MAPD < 0.25		Detection success rate	
			Number	Rate	Number	Rate
1	Embryo biopsy	2219	2207	99.5%	2206	99.4%
	NC and PC*	178	178	100.0%	178	100.0%
2	Embryo biopsy	1321	1319	99.8%	1318	99.8%
	NC and PC	126	126	100.0%	126	100.0%
3	Embryo biopsy	986	978	99.2%	975	98.9%
	NC and PC	98	98	100.0%	98	100.0%
4	Embryo biopsy	562	561	99.8%	558	99.3%
	NC and PC	52	52	100.0%	52	100.0%
5	Embryo biopsy	642	634	98.8%	632	98.4%
	NC and PC	80	80	100.0%	80	100.0%
Total	Embryo biopsy	5730	5699	99.5%	5689	99.3%
	NC and PC	534	534	100.0%	534	100.0%

*NC: Negative control samples. Each sample contained three euploid cells. PC: Positive control samples. Each sample contained three trisomy 21 cells

First, the sequencing data of the reference samples were analysed. The MAPD values for both negative and positive reference samples were all < 0.25, indicating that the sample testing processes at all centres met the required standards (Table 1). The MAPD values for all 5,730 embryo samples from patients were then analysed. 99.5% (5,699/5,730) of the embryos had MAPD values < 0.25, according to the results, and at least 98.8% of the embryos in each of the five centres had a MAPD < 0.25. The 31 samples with MAPD ≥ 0.25 were evenly distributed across the five centres. All embryos had a 99.3% (5689/5730) PGT-A/-SR detection success rate, with the lowest centre reaching 98.4% (632/642).

### Accuracy validation of PGT-A/-SR

To assess the accuracy of PGT-A/-SR in detecting CNVs in actual clinical practice, we collected the results of amniocentesis, neonatal blood, and abortions from patients who underwent single euploid blastocyst transfers. A total of 525 transfer cycles achieved ongoing pregnancies, with 458 transfer cycles with collected amniocentesis and neonatal blood results. The remaining 67 patients either declined amniocentesis detection, neonatal peripheral blood testing, or refused follow-up. The consistency with PGT-A negative results was 99.8% (457/458). One embryo had a discrepancy between the amniocentesis result and the PGT result; the karyotype of the amniotic fluid showed trisomy 21, while the PGT-A result indicated euploidy. In order to detect the patient’s chromosomal copy number, 60 samples were taken from various places on the placenta during the abortions. Forty-six samples had 20–30% mosaicism for chromosome 21, seven samples had no abnormalities in chromosome 21, five samples were suspected of maternal contamination during collection, and two samples failed quality control (Supplementary Table 2). This indicated that the trophectoderm cells of the embryo had low-level mosaicism, leading to a false negative result. 49 abortion samples were examined; none of them showed any abnormalities, hence the results were 100% consistent with negative PGT-A/-SR results (49/49). Using the results of 507 amniocentesis, newborn blood, and abortions as the gold standard, the overall consistency with PGT-A negative results was 99.8% (506/507).

Of the 3,609 embryos with PGT-A/-SR results indicating aneuploidy, 1,125 underwent the gold standard FISH testing. Chromosomes identified as aneuploid by PGT-A/-SR and high-risk chromosomes (chromosomes 13, 16, 18, 21, X, and Y) were analysed. A total of 4787 chromosomes were successfully detected by FISH. The data showed that 1,123 embryos (99.8%, 1,123/1,125) had FISH results consistent with the positive PGT-A/SR results. The PGT results of two embryos showed chromosomal abnormalities, while the FISH results indicated a diploid status (Supplementary Table 3).

Using 507 amniocentesis, neonatal blood, and miscarriage product results as the negative gold standard, and 1,125 embryo FISH results as the positive gold standard, PGT-A/-SR’s specificity, sensitivity, negative predictive value, and positive predictive value were 99.6%, 99.9%, 99.8% and 99.8%, respectively (Table 2).

**Table 2:** The performance of PGT-A/-SR

Performance of PGT-A/-SR	value	95% CI
Sensitivity (TP/(TP + FN))	99.9%(1123/1124)	99.5–100
Specificity (TN/(TN + FP))	99.6%(506/508)	98.6–99.9
Negative predictive value (TN/(TN + FN))	99.8%(506/507)	98.9–100
Positive predictive value (TP/(TP + FP))	99.8%(1123/1125)	99.4–100

### CNV analysis of embryos for PGT-A and PGT-SR

For 5,730 blastocysts from 1,015 couples, PGT-A/-SR was successfully performed on 5,689 embryos, resulting in a detection success rate of 99.3% (5,689/5,730). The euploidy rate was 36.6% (2,080/5,689). Considering that the cohort included both patients with normal and abnormal karyotypes, the euploidy rates for embryos from these two groups differed, and thus, the results were analysed separately.

2,150 embryos from 391 couples with normal karyotypes were evaluated; the detection success rate was 99.3% (2,135/2,150) and the euploidy rate was 45.9% (981/2,135). 3,580 embryos from 624 couples with abnormal karyotypes were analysed; the detection success rate was 99.3% (3,554/3,580) and the euploid + balanced rate was 30.9% (1,099/3,554) (Supplementary Table 4).

Stratified analysis of euploidy rates based on different morphological grades and embryonic days showed a decreasing trend in euploid + balanced rates from good (AA, AB, BA) to fair (BB) to poor (BC, CB, AC, CA, CC) embryos (PGT-A group: 53.0% vs. 47.8% vs. 40.0%, *p* = 0.001; PGT-SR group: 38.3% vs. 31.7% vs. 28.6%, *p* = 0.001). Good and fair embryos had higher euploid + balanced rates than poor embryos, and Day 5 blastocysts had higher euploidy rates than Day 6 blastocysts (PGT-A group: 47.7% vs. 41.8%, *p* = 0.013; PGT-SR group: 32.6% vs. 27.4%, *p* = 0.002) (Supplementary Table 5).

**Fig. 2:**
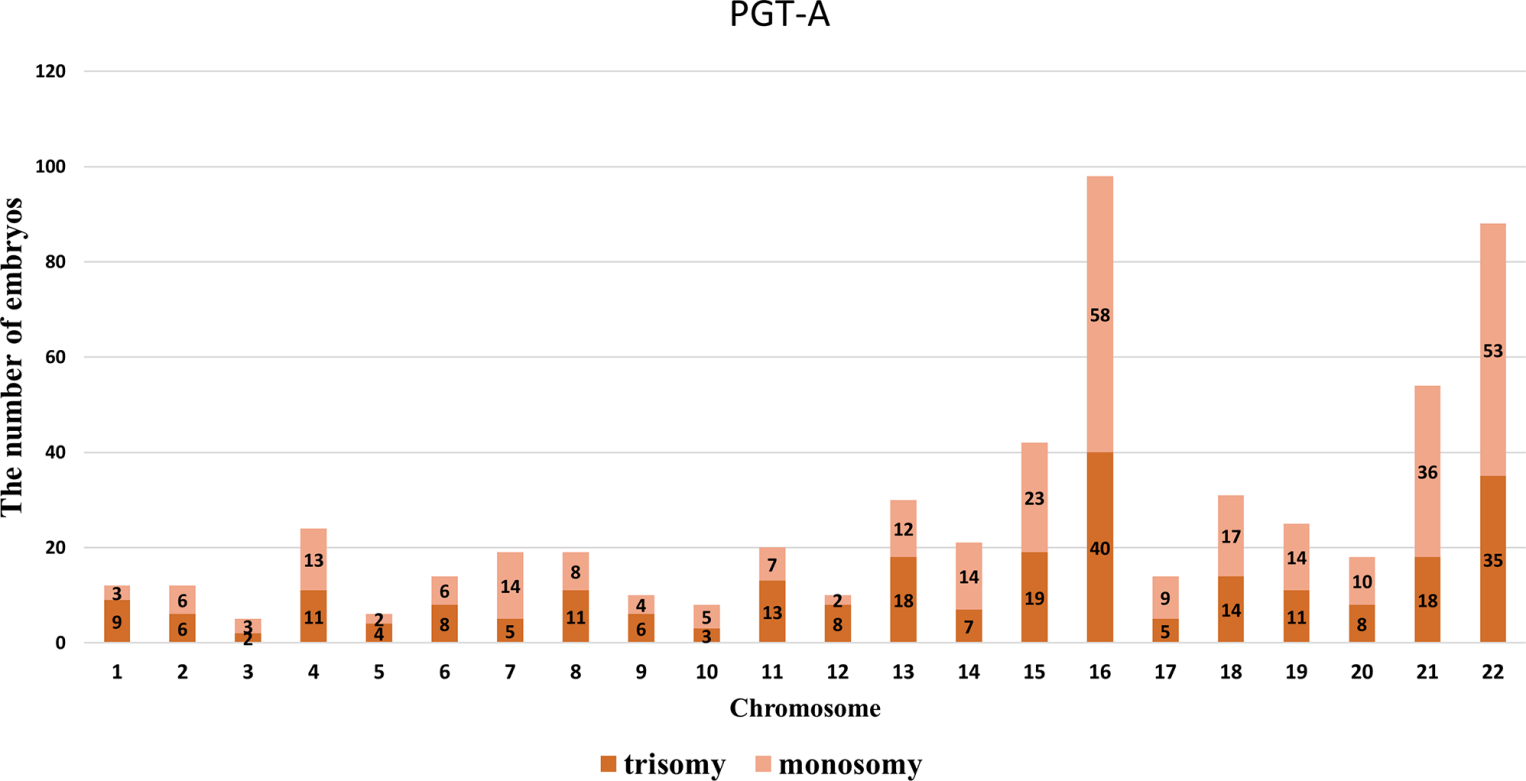
Number of embryos with trisomy and monosomy for each autosome in PGT-A couples with normal chromosomes. The numbers 1–22 correspond to autosomes 1–22

A total of 1,154 aneuploid embryos were produced from 391 couples with normal karyotypes. There were 580 autosomal monosomies and trisomies in all. Chromosomes 16, 22, 15, 13, and 21 were the most common autosomal trisomies, in decreasing order. Chromosomes 16, 22, 21, 15, and 18 had the highest frequency of autosomal monosomies, in decreasing order (Fig. 2). There were 2 embryos with X trisomy abnormalities and 21 embryos with X monosomy abnormalities.

**Fig. 3:**
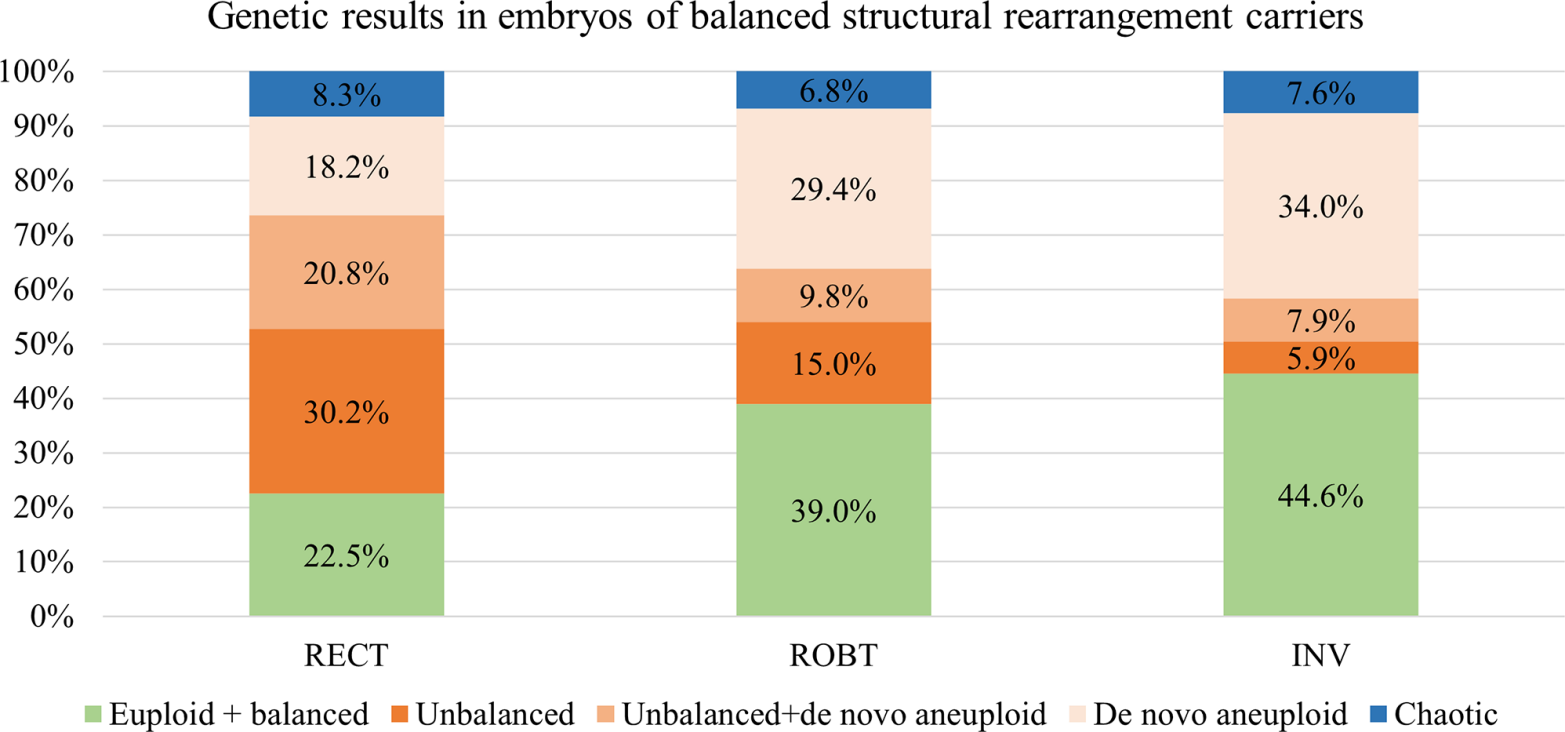
Proportion of (**a**) euploid + balanced, (**b**) unbalanced (unbalanced for SR but euploid for other non-rearranged chromosomes), (**c**) unbalanced + de novo aneuploid, (**d**) de novo aneuploid (diagnosed as aneuploid but balanced for the SR) and (**e**) chaotic (with more than five abnormalities). embryos in subgroups. RECT: Reciprocal Translocation Carriers. ROBT: Robertsonian Translocation Carriers. INV: Inversion Carriers

74 couples with complex structural rearrangements or sex chromosome abnormalities, including mosaicism, structural abnormalities, and numerical abnormalities, were excluded from the 624 couples with abnormal karyotypes. Of the remaining couples, 384 had reciprocal translocations (RecTs), 117 had Robertsonian translocations (RobTs), and 49 had non-polymorphic inversions. The number of embryos successfully tested in RecTs, RobTs and non-polymorphic inversions carriers was 2,143, 752, and 341, respectively. There were 483, 293, and 152 euploid + balanced embryos and 1,660, 459, and 189 aneuploid embryos, respectively. The euploid + balanced rate was highest among couples with non-polymorphic inversions (44.6%, 152/341), followed by those with RobTs (39.0%, 293/752), and lowest among those with RecTs (22.5%, 483/2,143). The aneuploid embryos were categorised based on the origin of the chromosomal abnormalities into four groups: euploid + balanced, unbalanced, unbalanced + de novo aneuploid, de novo aneuploid and chaotic [21]. Among inversion carriers, the proportion of embryos with de novo chromosomal abnormalities was the highest (34.0%), followed by RobT carriers (29.4%) and RecT carriers (18.2%) (Fig. 3).

According to the CNV study of de novo abnormal (including chaotic) embryos of those couples, the most prevalent autosomal trisomies were, in decreasing order, chromosomes 16, 22, 15, 13, and 6. Autosomal monosomies were most common on chromosomes 16, 22, 21, 15, and 13 (Supplementary Fig. 1). The top four trisomies and monosomies in abnormal embryos identified by PGT-A were consistent with these results.

### Clinical outcomes

Among the 1,015 couples, a total of 743 patients underwent 936 single blastocyst transfers. In the PGT-A group, there were 300 patients with a mean female age of 33.1 ± 4.0 years, and a total of 390 transfer cycles were performed. In the PGT-SR group, there were 443 patients with a mean female age of 29.4 ± 3.6 years, and a total of 546 transfer cycles were performed. The cumulative live birth rate was 66.0% (198/300) in the PGT-A group and 69.1% (306/443) in the PGT-SR group (Supplementary Table 6). The PGT-A and PGT-SR groups showed differences in female age, previous spontaneous miscarriages, previous failed transfer cycles, whether there were any abnormalities in previous pregnancies or miscarriages, and euploid + balanced rate. However, there were no significant differences in live birth rate for first transfer (*p* = 0.070) and cumulative live birth rate (*p* = 0.379) (Supplementary Table 6).

## Discussion

This was a large-scale, multicentre, real-world clinical study focusing on the populations with indications for PGT-A and PGT-SR. Firstly, the robustness of the ChromInst technology by analysing the MAPD values from embryo sequencing results across five reproductive centres were validated. Compared to traditional in vitro diagnostics testing, the workflow for PGT-A/-SR testing was significantly more complex, involving steps such as embryo culture, biopsy, whole-genome amplification, NGS sequencing, and result analysis. Biopsy samples from various reproductive centres may differ significantly because there are no established standards for these techniques. Osman and Scott et al. recommended a MAPD value of < 0.25 as a quality control standard. 99.5% (5,699/5,730) of the 5,730 embryos had MAPD values < 0.25, and the distribution of MAPD values among the samples from the five centres was highly similar. The five centres’ embryo detection success rates, which ranged from 98.4 to 99.8%, were similar. These indicated that the ChromInst may mitigate the variances in embryo samples brought about by variations in sampling and culture procedures, ensuring the stability and reliability of the sequencing results. PGT-A and PGT-SR based on the ChromInst protocol were well-suited for different centres.

Recent studies have shown that PGT-A based on whole-genome amplification using the MDA method may misdiagnose 17% of euploid embryos as aneuploid or with a high degree of mosaicism (> 50%) [29]. In this study, 1,125 embryos diagnosed as aneuploid by PGT-A/-SR were subjected to FISH, the gold standard for validation. Using FISH results as the positive gold standard, the concordance rate of positive PGT-A/-SR results was 99.8% (1,123/1,125), suggesting the ChromInst technique has excellent accuracy and low false-positive rates for PGT-A/-SR. In addition to false positives, false negatives should also be considered, embryos with false-negative results can lead to implantation failure, miscarriage, or fetal developmental abnormalities. To verify the accuracy of negative PGT-A/-SR results, we used CNV results from 507 cases of prenatal diagnosis, neonatal blood, and abortions as the negative gold standard. The concordance rate for negative PGT-A/-SR results was 99.8% (506/507). For 49 patients with miscarriages where the aborted embryos were euploid + balanced, the reason may be attributed to parental age, hormonal, immunological, and environmental factors [30–32]. There was one case of a false-negative result; however, a further analysis of samples taken from various parts of the miscarriage tissue showed that the TE cells had less than 30% low-level mosaicism. Popovic et al. conducted PGT-A testing on the inner cell mass (ICM) and trophectoderm (TE) cells of 34 donated embryos. Of the 18 embryos with euploid TE results in the first test, 16 had euploid ICM, while one showed mosaicism and another showed segmental abnormalities [33]. Due to the presence of mosaicism in embryos, PGT-A/-SR results may not always reflect the ICM status. However, based on large-scale clinical amniocentesis and miscarriage sample results, the discordance rate was only 0.2% (1/507). The validation of PGT-A/-SR results with such a large sample size is a highlight of our study.

Additionally, for individuals undergoing PGT-A and PGT-SR, the euploidy rate of embryos and the chromosomes with high abnormality rates are critical metrics. In our analysis of chromosomal abnormalities in embryos from the PGT-A cohort, the chromosomes most frequently associated with trisomy are16, 22, 15, 13, and 21, while those most frequently associated with monosomy are 16, 22, 21, 15, and 18. Monosomy X also has a relatively high frequency. Previous studies have reported that trisomy 16 and 22 are most frequent in trophoblast cells [19, 20]. In our study, trisomies of chromosomes 15, 13 and 21 were also found to be highly prevalent, in addition to the chromosomes 22 and 16. Zhang et al. conducted chromosomal analysis on 1,903 miscarriage samples and found high abnormality rates in chromosomes 16, 22, 21, X/Y, 13, and 15, with chromosomes 16, 22, 21, 15, and 13 being the top five in trisomic embryos [34]. This is consistent with the highly prevalent trisomic chromosomes observed in embryos. In the PGT-SR cohort, the proportion of embryos with unbalanced chromosomal constitutions was lowest for inversion carriers, followed by Robertsonian translocation carriers, and highest for reciprocal translocation carriers (47.8% vs. 54.2% vs. 69.2%) (Fig. 3). This aligns with previous studies [35, 36]. Although there might be differences due to maternal age, such that, Ogur et al. found transferable embryo rates of 35.8%, 33.2%, and 19.4% for inversion, Robertsonian, and reciprocal translocation carriers, respectively, which were lower than the rates reported in this study (44.6%, 39.0%, 22.5%) possibly due to difference in maternal age between cohorts [21]. Clinical genetic counselling for PGT-A/-SR patients benefited greatly from these useful findings.

In this study, the euploidy rate of embryos in the PGT-A group was 45.9% (981/2135), while in the PGT-SR group, it was 30.9% (1099/3554). Other studies have reported similar results, with euploidy rates ranging from 33.6 to 44.8% for PGT-A [37–39], and from 24.4 to 26.7% for PGT-SR cohorts [13, 40]. For these populations with high rates of aneuploid embryos, PGT-A/-SR can provide significant benefits by improving the success rate of each embryo transfer [41]. In a recent large-scale retrospective cohort study, the live birth rate in the PGT-A group, which included patients with indications such as advanced maternal age, recurrent miscarriage, fetal malformations or chromosomal abnormalities, and severe male factor infertility, was significantly higher than in the matched control group (52.6% vs. 34.2%) [42]. However, for non-indicated populations, such as younger women with a good prognosis, where the euploidy rate can be as high as 69.8%, PGT-A did not improve the live birth rate per transfer cycle, particularly the cumulative live birth rate [27]. For couples with chromosomal rearrangements, studies have reported a cumulative live birth rate of 55.8% [21]. In our study, the cumulative live birth rate in the PGT-SR group was 69.1% (306/443). The higher live birth rate may be associated with the younger age of the female partners in this cohort.

The study has several limitations. First, for patients with miscarriages where the abortions were normal, the cause of miscarriage could not be followed. Second, the technique cannot distinguish normal from balanced embryos, polyploidy, and contamination. This may lead to false-negative results in embryo testing. Third, we did not compare whole genome-amplified products across different platforms. The study results are applicable only to the ChromInst technology based on the MALBAC method.

## Conclusions

In conclusion, the study demonstrated that ChromInst-based PGT-A/-SR provided stable and accurate detection of embryonic CNVs, regardless of operational differences between clinical centres. For patients indicated for PGT-A/-SR, the ChromInst method can be clinically adopted for detecting embryonic chromosomal aneuploidy. The study also provided reliable data for genetic counseling prior to PGT-A/-SR by analysing the euploid + balanced rate and the frequency of aneuploidy in each chromosome.

## Electronic supplementary material

Below is the link to the electronic supplementary material.

Supplementary Material 1: Supplementary Fig. 1. Number of embryos with de novo autosome trisomy and monosomy in PGT-SR patients

Supplementary Material 2

## Data Availability

The data in this article will be shared on reasonable request to the corresponding author.

## Abbreviations

PGT-A: Preimplantation genetic testing for aneuploidy
PGT-SR: Preimplantation genetic testing for structural rearrangements
NGS: Next-generation sequencing
MAPD: Median absolute pairwise difference
IVF: In vitro fertilisation
SOP: Standard operating procedure
WGA: Whole genome amplification
FISH: Fluorescence in situ hybridisation
ICSI: Intracytoplasmic sperm injection
IVF-ET: In vitro fertilization-embryo transfer
CNV: Copy number variation
PPV: Positive predictive value
NPV: Negative predictive value
RecT: Reciprocal translocation
RobT: Robertsonian translocation

## References

[1] Rabinowitz M, Ryan A, Gemelos G, Hill M, Baner J, Cinnioglu C, et al. Origins and rates of aneuploidy in human blastomeres. Fertil Steril. 2012;97:395–401. 10.1016/j.fertnstert.2011.11.034. 22195772 10.1016/j.fertnstert.2011.11.034

[2] Scott RT, Ferry K, Su J, Tao X, Scott K, Treff NR. Comprehensive chromosome screening is highly predictive of the reproductive potential of human embryos: a prospective, blinded, nonselection study. Fertil Steril. 2012;97:870–5. 10.1016/j.fertnstert.2012.01.104. 22305103 10.1016/j.fertnstert.2012.01.104

[3] Coticchio G, Dal Canto M, Mignini Renzini M, Guglielmo MC, Brambillasca F, Turchi D, et al. Oocyte maturation: gamete-somatic cells interactions, meiotic resumption, cytoskeletal dynamics and cytoplasmic reorganization. Hum Reprod Update. 2015;21:427–54. 10.1093/humupd/dmv011. 25744083 10.1093/humupd/dmv011

[4] Wang WH, Sun QY. Meiotic spindle, spindle checkpoint and embryonic aneuploidy. Front Biosci. 2006;11:620–36. 10.2741/1822. 16146756 10.2741/1822

[5] McCoy RC, Summers MC, McCollin A, Ottolini CS, Ahuja K, Handyside AH. Meiotic and mitotic aneuploidies drive arrest of in vitro fertilized human preimplantation embryos. Genome Med. 2023;15:77. 10.1186/s13073-023-01231-1. 37779206 10.1186/s13073-023-01231-1 PMC10544495

[6] Verdyck P, Altarescu G, Santos-Ribeiro S, Vrettou C, Koehler U, Griesinger G, et al. Aneuploidy in oocytes from women of advanced maternal age: analysis of the causal meiotic errors and impact on embryo development. Hum Reprod. 2023;38:2526–35. 10.1093/humrep/dead201. 37814912 10.1093/humrep/dead201

[7] Fu W, Cui Q, Yang Z, Bu Z, Shi H, Bi B, et al. High sperm DNA fragmentation increased embryo aneuploidy rate in patients undergoing preimplantation genetic testing. Reprod Biomed Online. 2023;47:103366. 10.1016/j.rbmo.2023.103366. 37812976 10.1016/j.rbmo.2023.103366

[8] Munné S, Sandalinas M, Escudero T, Fung J, Gianaroli L, Cohen J. Outcome of preimplantation genetic diagnosis of translocations. Fertil Steril. 2000;73:1209–18. 10.1016/s0015-0282(00)00495-7. 10856485 10.1016/s0015-0282(00)00495-7

[9] Capalbo A, Rienzi L, Cimadomo D, Maggiulli R, Elliott T, Wright G, et al. Correlation between standard blastocyst morphology, euploidy and implantation: an observational study in two centers involving 956 screened blastocysts. Hum Reprod. 2014;29:1173–81. 10.1093/humrep/deu033. 24578475 10.1093/humrep/deu033

[10] Rienzi L, Capalbo A, Stoppa M, Romano S, Maggiulli R, Albricci L, et al. No evidence of association between blastocyst aneuploidy and morphokinetic assessment in a selected population of poor-prognosis patients: a longitudinal cohort study. Reprod Biomed Online. 2015;30:57–66. 10.1016/j.rbmo.2014.09.012. 25458852 10.1016/j.rbmo.2014.09.012

[11] Coonen E, Rubio C, Christopikou D, Dimitriadou E, Gontar J, Goossens V, et al. ESHRE PGT Consortium good practice recommendations for the detection of structural and numerical chromosomal aberrations. Hum Reprod Open. 2020;2020:hoaa017. 10.1093/hropen/hoaa017. 32500102 10.1093/hropen/hoaa017 PMC7257111

[12] Tian Y, Li M, Yang J, Chen H, Lu D. Preimplantation genetic testing in the current era, a review. Arch Gynecol Obstet. 2024. 10.1007/s00404-024-07370-z. 38376520 10.1007/s00404-024-07370-z

[13] Huang C, Jiang W, Zhu Y, Li H, Lu J, Yan J, et al. Pregnancy outcomes of reciprocal translocation carriers with two or more unfavorable pregnancy histories: before and after preimplantation genetic testing. J Assist Reprod Genet. 2019;36:2325–31. 10.1007/s10815-019-01585-9. 31522368 10.1007/s10815-019-01585-9 PMC6885462

[14] Osman EK, Neal SA, Tiegs AW, Hanson BM, Kim JG, Franasiak JM, et al. Consistency in rates of diagnosis of embryonic mosaicism, segmental abnormalities, and no call results among experienced embryologists performing preimplantation genetic testing for aneuploidy. F S Rep. 2020;1:119–24. 10.1016/j.xfre.2020.05.005. 34223227 10.1016/j.xfre.2020.05.005 PMC8244265

[15] Gao FF, Chen L, Bo SP, Yao YX, Xu ZL, Ding QY, et al. ChromInst: a single cell sequencing technique to accomplish pre-implantation comprehensive chromosomal screening overnight. PLoS ONE. 2021;16:e0251971. 10.1371/journal.pone.0251971. 34015059 10.1371/journal.pone.0251971 PMC8136696

[16] Chen L, Sun Q, Xu J, Fu H, Liu Y, Yao Y, et al. A non-invasive chromosome screening strategy for prioritizing in vitro fertilization embryos for implantation. Front Cell Dev Biol. 2021;9:708322. 10.3389/fcell.2021.708322. 34434931 10.3389/fcell.2021.708322 PMC8380813

[17] Girardi L, Figliuzzi M, Poli M, Serdarogullari M, Patassini C, Caroselli S, et al. The use of copy number loads to designate mosaicism in blastocyst stage PGT-A cycles: fewer is better. Hum Reprod. 2023;38:982–91. 10.1093/humrep/dead049. 36928183 10.1093/humrep/dead049

[18] Zhai F, Wang Y, Li H, Wang Y, Zhu X, Kuo Y, et al. Preimplantation genetic testing for structural rearrangement based on low-coverage next-generation sequencing accurately discriminates between normal and carrier embryos for patients with translocations. Reprod Biomed Online. 2022;45:473–80. 10.1016/j.rbmo.2022.05.012. 35780041 10.1016/j.rbmo.2022.05.012

[19] Benn P, Grati FR. Aneuploidy in first trimester chorionic villi and spontaneous abortions: Windows into the origin and fate of aneuploidy through embryonic and fetal development. Prenat Diagn. 2021;41:519–24. 10.1002/pd.5795. 32677063 10.1002/pd.5795

[20] Shen J, Wu W, Gao C, Ochin H, Qu D, Xie J, et al. Chromosomal copy number analysis on chorionic villus samples from early spontaneous miscarriages by high throughput genetic technology. Mol Cytogenet. 2016;9:7. 10.1186/s13039-015-0210-z. 26819630 10.1186/s13039-015-0210-z PMC4728779

[21] Ogur C, Kahraman S, Griffin DK, Cinar Yapan C, Tufekci MA, Cetinkaya M, et al. PGT for structural chromosomal rearrangements in 300 couples reveals specific risk factors but an interchromosomal effect is unlikely. Reprod Biomed Online. 2023;46:713–27. 10.1016/j.rbmo.2022.07.016. 36803887 10.1016/j.rbmo.2022.07.016

[22] Schoolcraft WB, Gardner DK, Lane M, Schlenker T, Hamilton F, Meldrum DR. Blastocyst culture and transfer: analysis of results and parameters affecting outcome in two in vitro fertilization programs. Fertil Steril. 1999;72:604–9. 10.1016/s0015-0282(99)00311-8. 10521095 10.1016/s0015-0282(99)00311-8

[23] Zhang S, Xie P, Lan F, Yao Y, Ma S, Hu L, et al. Conventional IVF is feasible in preimplantation genetic testing for aneuploidy. J Assist Reprod Genet. 2023;40:2333–42. 10.1007/s10815-023-02916-7. 37656381 10.1007/s10815-023-02916-7 PMC10504148

[24] Tan YQ, Tan K, Zhang SP, Gong F, Cheng DH, Xiong B, et al. Single-nucleotide polymorphism microarray-based preimplantation genetic diagnosis is likely to improve the clinical outcome for translocation carriers. Hum Reprod. 2013;28:2581–92. 10.1093/humrep/det271. 23847111 10.1093/humrep/det271

[25] Zhang S, Luo K, Cheng D, Tan Y, Lu C, He H, et al. Number of biopsied trophectoderm cells is likely to affect the implantation potential of blastocysts with poor trophectoderm quality. Fertil Steril. 2016;105:1222. 7.e4. 26820770 10.1016/j.fertnstert.2016.01.011

[26] Harton GL, Harper JC, Coonen E, Pehlivan T, Vesela K, Wilton L. ESHRE PGD consortium best practice guidelines for fluorescence in situ hybridization-based PGD. Hum Reprod. 2011;26:25–32. 10.1093/humrep/deq230. 20966461 10.1093/humrep/deq230

[27] Yan J, Qin Y, Zhao H, Sun Y, Gong F, Li R, et al. Live birth with or without preimplantation genetic testing for Aneuploidy. N Engl J Med. 2021;385:2047–58. 10.1056/NEJMoa2103613. 34818479 10.1056/NEJMoa2103613

[28] Osman E, Neal K, Tiegs SA, Hanson AW, Kim BM, Franasiak JG. Consistency in rates of diagnosis of embryonic mosaicism, segmental abnormalities, and no call results among experienced embryologists performing preimplantation genetic testing for aneuploidy. F S Rep. 2020;1:119–24. 10.1016/j.xfre.2020.05.005. 34223227 10.1016/j.xfre.2020.05.005 PMC8244265

[29] Shen X, Chen D, Ding C, Xu Y, Fu Y, Cai B, et al. Evaluating the application value of NGS-based PGT-A by screening cryopreserved MDA products of embryos from PGT-M cycles with known transfer outcomes. J Assist Reprod Genet. 2022;39:1323–31. 10.1007/s10815-022-02447-7. 35275308 10.1007/s10815-022-02447-7 PMC9174381

[30] Magnus MC, Wilcox AJ, Morken NH, Weinberg CR, Håberg SE. Role of maternal age and pregnancy history in risk of miscarriage: prospective register based study. BMJ. 2019;364:l869. 10.1136/bmj.l869. 30894356 10.1136/bmj.l869 PMC6425455

[31] Garrido-Gimenez C, Alijotas-Reig J. Recurrent miscarriage: causes, evaluation and management. Postgrad Med J. 2015;91:151–62. 10.1136/postgradmedj-2014-132672. 25681385 10.1136/postgradmedj-2014-132672

[32] Agenor A, Bhattacharya S. Infertility and miscarriage: common pathways in manifestation and management. Womens Health (Lond). 2015;11:527–41. 10.2217/whe.15.19. 26238301 10.2217/whe.15.19

[33] Popovic M, Dheedene A, Christodoulou C, Taelman J, Dhaenens L, Van Nieuwerburgh F, et al. Chromosomal mosaicism in human blastocysts: the ultimate challenge of preimplantation genetic testing? Hum Reprod. 2018;33:1342–54. 10.1093/humrep/dey106. 29796631 10.1093/humrep/dey106

[34] Zhang J, Mu F, Guo Z, Cai Z, Zeng X, Du L, et al. Chromosome analysis of foetal tissue from 1903 spontaneous abortion patients in 5 regions of China: a retrospective multicentre study. BMC Pregnancy Childbirth. 2023;23:818. 10.1186/s12884-023-06108-0. 38007414 10.1186/s12884-023-06108-0 PMC10675863

[35] Zhou F, Ren J, Li Y, Keqie Y, Peng C, Chen H, et al. Preimplantation genetic testing in couples with balanced chromosome rearrangement: a four-year period real world retrospective cohort study. BMC Pregnancy Childbirth. 2024;24:86. 10.1186/s12884-023-06237-6. 38280990 10.1186/s12884-023-06237-6 PMC10821259

[36] Yuan P, Zheng L, Ou S, Zhao H, Li R, Luo H, et al. Evaluation of chromosomal abnormalities from preimplantation genetic testing to the reproductive outcomes: a comparison between three different structural rearrangements based on next-generation sequencing. J Assist Reprod Genet. 2021;38:709–18. 10.1007/s10815-020-02053-5. 33409753 10.1007/s10815-020-02053-5 PMC7910334

[37] La Marca A, Capuzzo M, Longo M, Imbrogno MG, Spedicato GA, Fiorentino F, et al. The number and rate of euploid blastocysts in women undergoing IVF/ICSI cycles are strongly dependent on ovarian reserve and female age. Hum Reprod. 2022;37:2392–401. 10.1093/humrep/deac191. 36006017 10.1093/humrep/deac191

[38] Tong J, Niu Y, Wan A, Zhang T. Comparison of day 5 blastocyst with day 6 blastocyst: evidence from NGS-based PGT-A results. J Assist Reprod Genet. 2022;39:369–77. 10.1007/s10815-022-02397-0. 35013836 10.1007/s10815-022-02397-0 PMC8956767

[39] Munné S, Kaplan B, Frattarelli JL, Child T, Nakhuda G, Shamma FN, et al. Preimplantation genetic testing for aneuploidy versus morphology as selection criteria for single frozen-thawed embryo transfer in good-prognosis patients: a multicenter randomized clinical trial. Fertil Steril. 2019;112:1071. 9.e7. 31551155 10.1016/j.fertnstert.2019.07.1346

[40] Insogna IG, Lanes A, Dobson L, Ginsburg ES, Racowsky C, Yanushpolsky E. Blastocyst conversion rate and ploidy in patients with structural rearrangements. J Assist Reprod Genet. 2021;38:1143–51. 10.1007/s10815-021-02131-2. 33656620 10.1007/s10815-021-02131-2 PMC8190241

[41] Wang S, Liu L, Ma M, Wang H, Han Y, Guo X, et al. Preimplantation genetic testing for aneuploidy helps to achieve a live birth with fewer transfer cycles for the blastocyst FET patients with unexplained recurrent implantation failure. Arch Gynecol Obstet. 2023;308:599–610. 10.1007/s00404-023-07041-5. 37246978 10.1007/s00404-023-07041-5

[42] Ma S, Liao J, Zhang S, Yang X, Hocher B, Tan J, et al. Exploring the efficacy and beneficial population of preimplantation genetic testing for aneuploidy start from the oocyte retrieval cycle: a real-world study. J Transl Med. 2023;21:779. 10.1186/s12967-023-04641-2. 37919732 10.1186/s12967-023-04641-2 PMC10623718

