# Supplementary figures and images for: ChromInst: a multicentre evaluation of robustness in aneuploidy and structural rearrangement testing
Source: J Transl Med. 2025 Feb 26;23:230. doi: 10.1186/s12967-025-06242-7 (PMC11863937; doi:10.1186/s12967-025-06242-7)

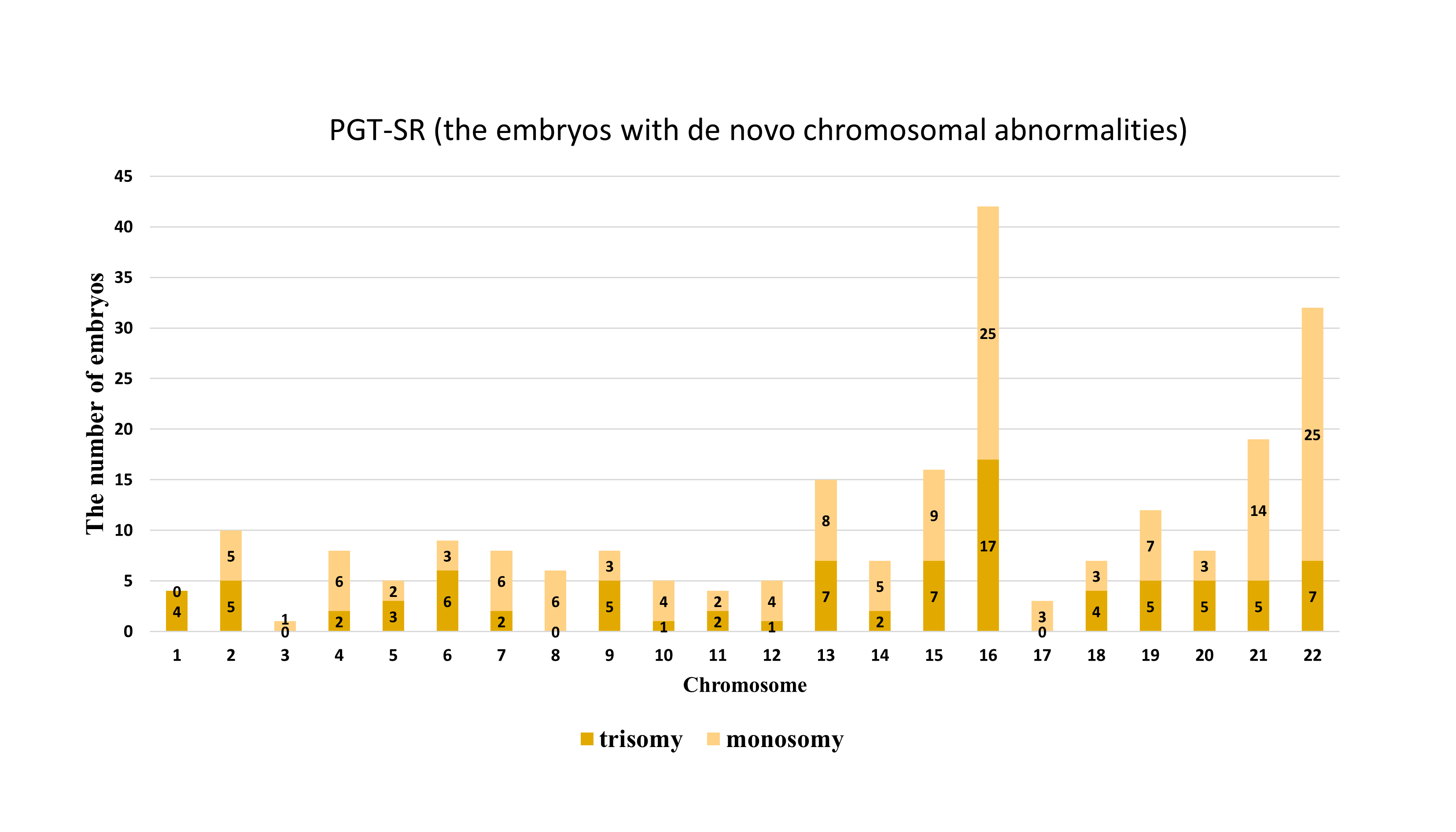

Supplement: Supplementary file 1 — Supplementary Material 1: Supplementary Fig. 1. Number of embryos with de novo autosome trisomy and monosomy in PGT-SR patients [file 12967_2025_6242_MOESM1_ESM.tif]
