# Supplementary material for: ChromInst: a multicentre evaluation of robustness in aneuploidy and structural rearrangement testing
Source: J Transl Med. 2025 Feb 26;23:230. doi: 10.1186/s12967-025-06242-7 (PMC11863937; doi:10.1186/s12967-025-06242-7)
Supplement: Supplementary file 2 — Supplementary Material 2 [file 12967_2025_6242_MOESM2_ESM.docx]

**Supplementary Tables**

**Supplementary Table 1.** Embryo culture and biopsy protocols used in the clinical centres.

| Centre | Embryo culture medium | The brand of Laser system | The power of Laser system | Time of embryo culture | Biopsy sample rinse | Number of biopsy technicians |
| --- | --- | --- | --- | --- | --- | --- |
| 01 | Vitrolife | RI | 360mW | D4 | None | 3 |
| 02 | Vitrolife | OCTAX | 150mW | D5 | MOPS (Vitrolife) | 2 |
| 03 | Vitrolife | RI | 360mW | D4 | PBS | 2 |
| 04 | COOK | Hamilton | 300mW | D5 | None | 3 |
| 05 | Quinn's | Hamilton | 300mW | D5 | None | 2 |

Centre 01-05: refer to five reproductive medicine centres.

**Supplementary Table 2.** NGS results of placental samples.

| **NGS Results** | **Sample size** | **Example of NGS results** |
| --- | --- | --- |
| 20%-30% mosaic trisomy 21 | 46 | 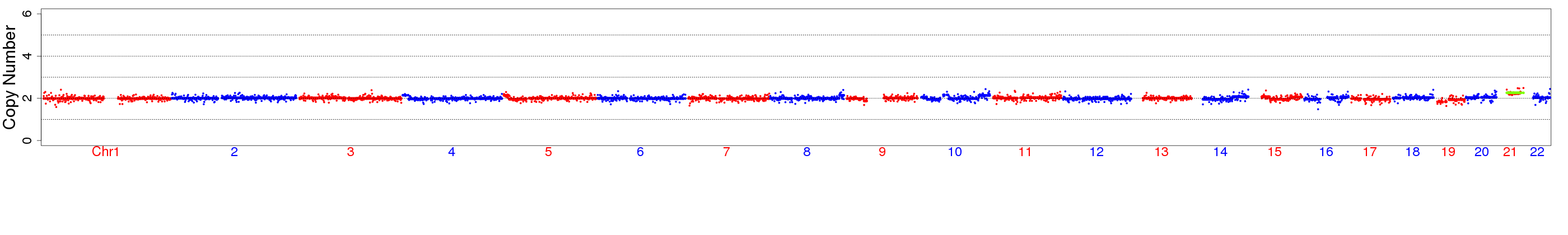 |
| Euploid chromosome 21 | 7 | 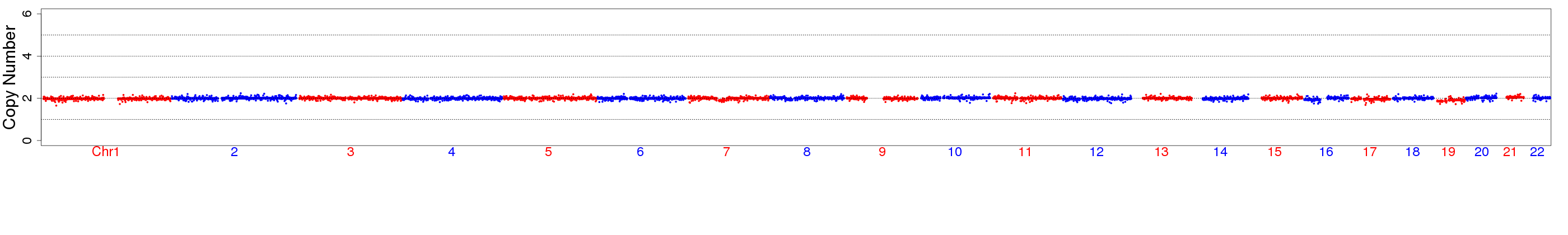 |

**Supplementary Table 3.** The number of chromosomes tested by FISH.

| **Chromosomes** | **Number of FISH tested chromosomes** | **Probe (Vysis)** | **Inconsistent results** |
| --- | --- | --- | --- |
| 1 | 27 | 1q |  |
| 2 | 35 | 2q |  |
| 3 | 18 | 3q |  |
| 4 | 48 | 4q |  |
| 5 | 20 | 5p; 5q |  |
| 6 | 29 | CEP 6; 6q |  |
| 7 | 46 | 7g |  |
| 8 | 34 | 8p; 8q |  |
| 9 | 27 | 9p; 9q |  |
| 10 | 23 | 10q |  |
| 11 | 33 | 11q |  |
| 12 | 18 | 12q; 12p |  |
| 13 | 847 | 13q14 |  |
| 14 | 117 | 14q |  |
| 15 | 94 | CEP 15 |  |
| 16 | 864 | CEP 16 | One embry: FISH-negative; PGT-postive |
| 17 | 24 | 17q |  |
| 18 | 912 | 18p; CEP 18 | One embry: FISH-negative; PGT-postive |
| 19 | 42 | 19q |  |
| 20 | 33 | 20q; 20p |  |
| 21 | 430 | 21q |  |
| 22 | 169 | 22q |  |
| X/Y | 897 | CEP X; CEP Y |  |
| Total | 4787 |  |  |

**Supplementary Table 4.** Baseline characteristics of all PGT-A and PGT-SR patients.

|  | PGT-A | PGT-SR | P value |
| --- | --- | --- | --- |
| No. of patients | 391 | 624 |  |
| Female Age (y) | 33.8 ± 4.4 | 29.7 ± 3.7 | <0.001 |
| No. of previous failed transfer cycles |  |  | <0.001 |
| 0 | 82.6%(323/391) | 96.8%(604/624) |  |
| 1-2 | 5.6%(22/391) | 2.4%(15/624) |  |
| ≥3 | 11.8%(46/391) | 0.8%(5/624) |  |
| No. of previous early spontaneous miscarriages |  |  | <0.001 |
| 0 | 19.2%(75/391) | 66.5%(415/624) |  |
| 1 | 4.3%(17/391) | 6.7%(42/624) |  |
| ≥2 | 76.5%(299/391) | 26.8%(167/624) |  |
| Having a child with chromosomal abnormalities or chromosomal abnormalities detected in chorionic villus tissue following a miscarriage |  |  | <0.001 |
| Yes | 45.3%(177/391) | 6.4%(40/624) |  |
| No | 54.7%(214/391) | 93.6%(584/624) |  |
| Severe teratozoospermia |  |  | 0.518 |
| Yes | 4.1%(16/391) | 5%(31/624) |  |
| No | 95.9%(375/391) | 95%(593/624) |  |
| Total number of blastocysts | 2150 | 3580 |  |
| No. of blastocysts | 5.5 ± 3.4 | 5.7± 3.6 | 0.294 |
| PGT detection success rate | 99.3% (2,135/2,150) | 99.3% (3,554/3,580) | 0.901 |
| Euploid + balanced rate | 45.9% (981/2,135) | 30.9%(1,099/3,554) | <0.001 |

**Supplementary Table 5.** Euploidy rates of embryos with different morphological grades and days of blastocysts.

| Groups | Subgroups | Proportion | Euploid + balanced rates | *P* value |
| --- | --- | --- | --- | --- |
| PGT-A group: | Morphological grades |  |  |  |
|  | Good^#^ | 10.3%(219/2135) | 53.0%(116/219) | 0.001 |
|  | Fair | 54.5%(1163/2135) | 47.8%(556/1163) |  |
|  | Poor | 35.3%(753/2135) | 40.0%(309/753) |  |
|  | Days of blastocysts |  |  |  |
|  | D5 | 71.4%(1524/2135) | 47.7%(727/1524) | 0.013 |
|  | D6 | 28.5%(608/2135) | 41.8%(254/608) |  |
|  | D7 | 0.1%(3/2135) | 0%(0/3) |  |
| PGT-SR group: | Morphological grades |  |  |  |
|  | Good | 10.4%(371/3554) | 38.3%(142/371) | 0.001 |
|  | Fair | 43.6%(1551/3554) | 31.7%(491/1551) |  |
|  | Poor | 45.9%(1632/3554) | 28.6%(466/1632) |  |
|  | Days of blastocysts |  |  |  |
|  | D5 | 67.2%(2388/3554) | 32.6%(779/2388) | 0.002 |
|  | D6 | 32.8%(1166/3554) | 27.4%(320/1166) |  |
|  | D7 | 0%(0/3554) | / |  |

^#^Good: morphological grades were AA, AB, BA. Fair: morphological grades were BB. Poor: morphological grades were AC, CA, BC, CB, CC.

**Supplementary Table 6.** The baseline characteristics and clinical outcomes of PGT-A and PGT-SR patients who have undergone single embryo transfer.

|  | **PGT-A** | **PGT-SR** | **P value** |
| --- | --- | --- | --- |
| No. of patients | 300 | 443 |  |
| Female Age (y) | 33.1 ± 4.0 | 29.4 ± 3.6 | <.001 |
| No. of previous failed transfer cycles |  |  | <.001 |
| 0 | 84.33(253/300) | 97.29(431/443) |  |
| 1-2 | 4.67(14/300) | 2.26(10/443) |  |
| ≥3 | 11.00(33/300) | 0.45(2/443) |  |
| No. of previous early spontaneous miscarriages |  |  | <.001 |
| 0 | 12.00(36/300) | 74.49(330/443) |  |
| 1 | 85.33(256/300) | 25.51(113/443) |  |
| ≥2 | 2.67(8/300) | 0.00(431/443) |  |
| Having a child with chromosomal abnormalities or chromosomal abnormalities detected in chorionic villus tissue following a miscarriage |  |  | <.001 |
| Yes | 47.33(142/300) | 5.42(24/443) |  |
| No | 52.67(158/300) | 94.58(419/443) |  |
| Severe teratozoospermia |  |  | 0.479 |
| Yes | 3.67(11/300) | 4.74(21/443) |  |
| No | 96.33(289/300) | 95.26(422/443) |  |
| Total number of blastocysts | 1828 | 2833 |  |
| No. of blastocysts | 6.09 ± 3.48 | 6.40 ± 3.83 | 0.275 |
| PGT detection success rate | 99.3%(1815/1828) | 99.2%(2811/2833) | 0.801 |
| Euploid + balanced rate | 51.9%(942/1815) | 38.0%(1069/2811) | <.001 |
| Clinical pregnancy rate for first transfer | 65.00(195/300) | 68.40(303/443) | 0.334 |
| Miscarriage rate for first transfer | 14.9%(29/195) | 10.2%(31/303) | 0.120 |
| Ongoing pregnancy rate for first transfer | 55.3%(166/300) | 60.9%(270/443)* | 0.127 |
| Live birth rate for first transfer | 53.3%(160/300) | 60.0%(266/443) | 0.070 |
| No. of all transfer cycles | 390 | 546 |  |
| Cumulative live birth rate per patients (Multiple embryo transfers ) | 66.0% (198/300) | 69.1% (306/443) | 0.379 |

No.: number. * Two patients were lost to follow-up during the ongoing pregnancy visits.
